# Author Correction: Systematic protein-protein interaction mapping for clinically relevant human GPCRs

**DOI:** 10.1038/s44320-024-00080-3

**Published:** 2025-01-06

**Authors:** Kate Sokolina, Saranya Kittanakom, Jamie Snider, Max Kotlyar, Pascal Maurice, Jorge Gandía, Abla Benleulmi-Chaachoua, Kenjiro Tadagaki, Atsuro Oishi, Victoria Wong, Ramy H Malty, Viktor Deineko, Hiroyuki Aoki, Shahreen Amin, Zhong Yao, Xavier Morató, David Otasek, Hiroyuki Kobayashi, Javier Menendez, Daniel Auerbach, Stephane Angers, Natasa Pržulj, Michel Bouvier, Mohan Babu, Francisco Ciruela, Ralf Jockers, Igor Jurisica, Igor Stagljar

**Affiliations:** 1 Donnelly Centre, University of Toronto, Toronto, M5S 3E1 Canada; 2 Princess Margaret Cancer Centre, University Health Network, University of Toronto, Toronto, M5G 1L7 Canada; 3 Inserm, U1016, Institut Cochin, Paris, France; 4 CNRS UMR 8104, Paris, France; 5 Univ. Paris Descartes, Sorbonne Paris Cité, Paris, France; 6 Unitat de Farmacologia, Departament de Patologia i Terapèutica Experimental, Facultat de Medicina, IDIBELL, Universitat de Barcelona, L’Hospitalet de Llobregat, Barcelona, 08901 Spain; 7 Institut de Neurociències, Universitat de Barcelona, Barcelona, 08901 Spain; 8 Department of Biochemistry, Research and Innovation Centre, University of Regina, Regina, S4S 0A2 Canada; 9 Department of Biochemistry, Institute for Research in Immunology & Cancer, Université de Montréal, Montréal, H3T 1J4 Canada; 10 Dualsystems Biotech AG, Schlieren, 8952 Switzerland; 11 Department of Pharmaceutical Sciences, Leslie Dan Faculty of Pharmacy and Department of Biochemistry, Faculty of Medicine, University of Toronto, Toronto, M5S 3M2 Canada; 12 Department of Computing, University College London, London, WC1E 6BT UK; 13 Departments of Medical Biophysics and Computer Science, University of Toronto, Toronto, M5G 2M9 Canada; 14 Institute of Neuroimmunology, Slovak Academy of Sciences, Bratislava, Slovakia; 15 Department of Molecular Genetics, University of Toronto, Toronto, M5S 1A8 Canada; 16 Department of Biochemistry, University of Toronto, Toronto, M5S 1A8 Canada

## Abstract

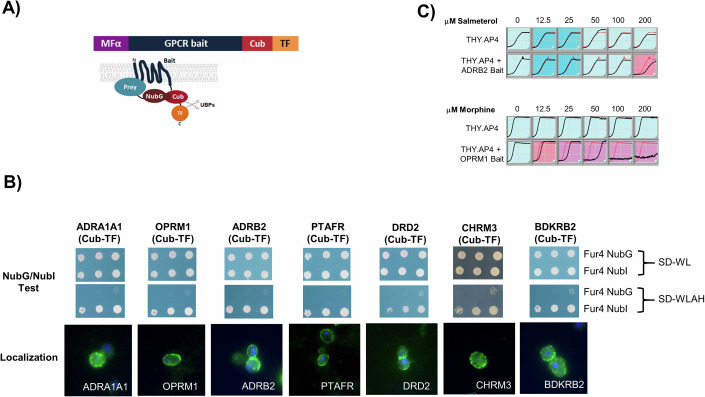

**Correction to:** *Molecular Systems Biology* (2017) 13:918. 10.15252/msb.20167430 | Published online 6 January 2025

The journal contacted the authors after being made aware of irregularities within **Fig. 2B**. Based on the exchanges with the authors, the journal has agreed to retract and replace the following figure panel.
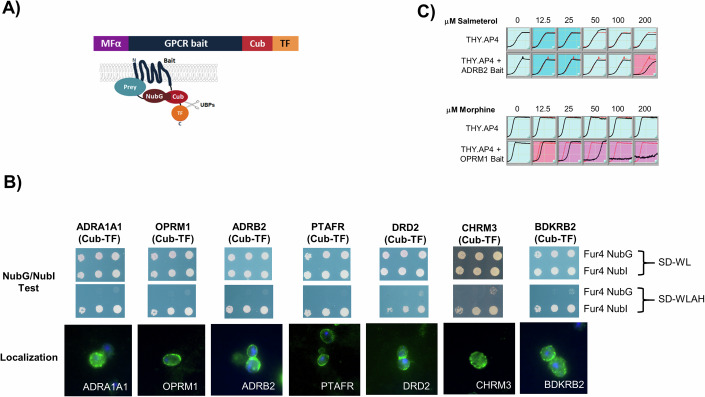


**Figure 2. Original.**

Figure 2B is retracted and replaced. Source data for Figure 2B is published with this correction.

Author statement:

The image being shown in **Fig. 2B** of the published paper for the ADRA1A1 NubG/NubI test is a duplicate of the corresponding image for OPRM1 (shown next to ADRA1A1 in the figure). This was a copy/paste error when assembling the images during figure creation.

The figure is updated with the correct image for ADRA1A1.

These errors do not affect the conclusions of the original paper.

All authors agree with this corrigendum.

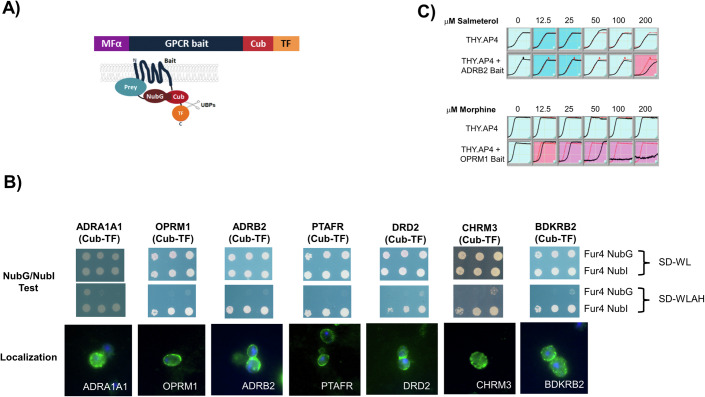



**Figure 2. Corrected.**

## Supplementary information

Full ADR1A1 Sample Images on Plates

Source Plate Images

