# Supplementary material for: Systematic protein–protein interaction mapping for clinically relevant human GPCRs
Source: Mol Syst Biol. 2017 Mar 15;13(3):918. doi: 10.15252/msb.20167430 (PMC5371730; doi:10.15252/msb.20167430)
Supplement: Supplementary file 1 — Expanded View Figures PDF [file MSB-13-918-s001.pdf]

Expanded View Figures

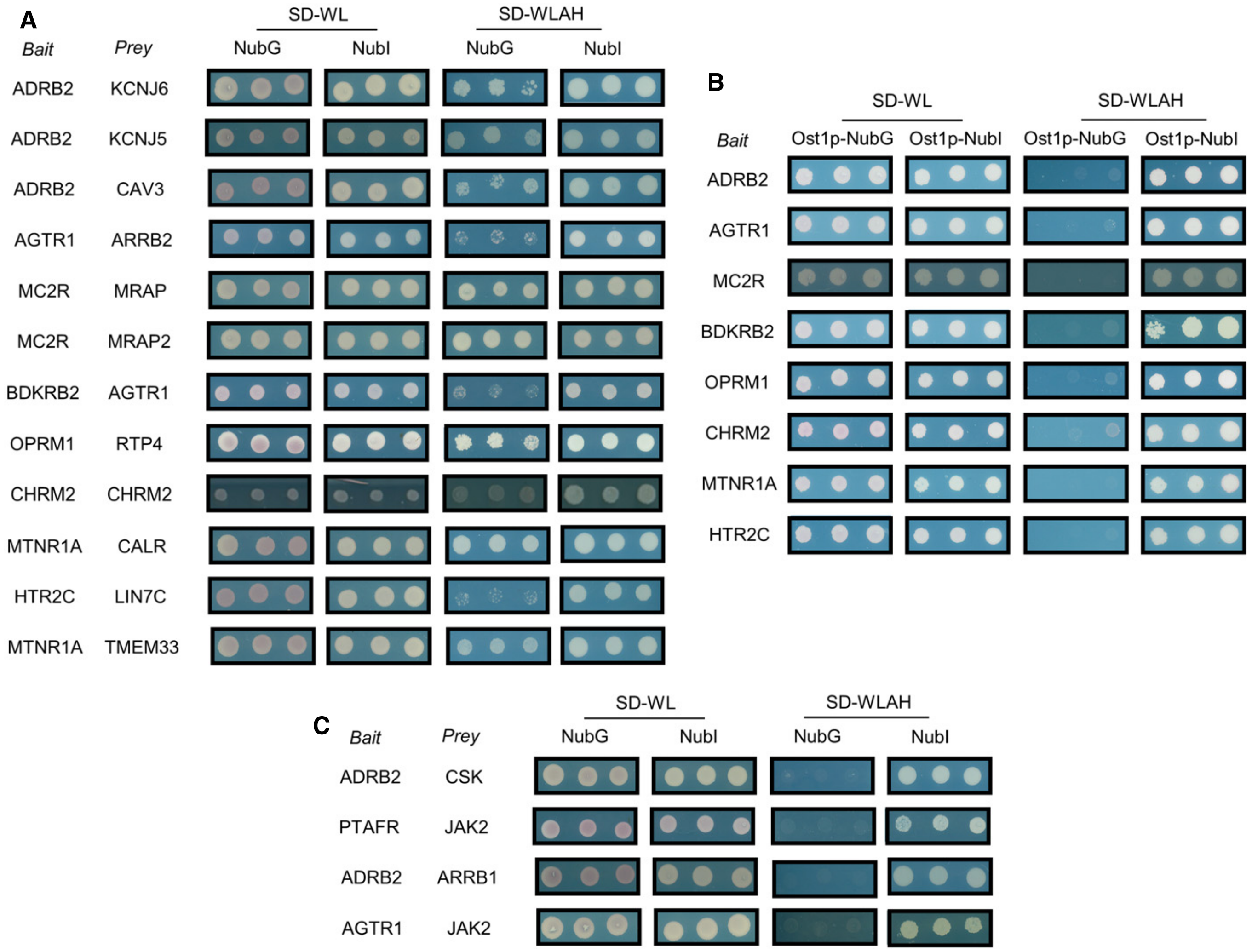

Figure EV1. Validation of MYTH baits using known interactions.

A GPCR interactions confirmed by MYTH. Yeast cells were co-transfected with GPCR bait and selected preys corresponding to previously identified interaction partners. Cells were spotted in triplicate on SD-WL media (which selects only for presence of bait and prey plasmid) and SD-WLAH media (which selects for interaction between bait and prey). Growth of cells on SD-WLAH media using NubI constructs confirmed expression of prey (i.e. since NubI leads to reconstitution of split-ubiquitin/reporter activation in the absence of a bait–prey interaction).

B GPCR baits tested as above but using the non-interacting control prey Ost1p. The lack of interaction with Ost1p-NubG on SD-WLAH media demonstrates the specificity of the bait interactions with preys demonstrated above.

C Representative sample of previously identified GPCR interactions tested in MYTH but which could not be confirmed (see Table EV2 for complete list).

 © 2017 The Authors

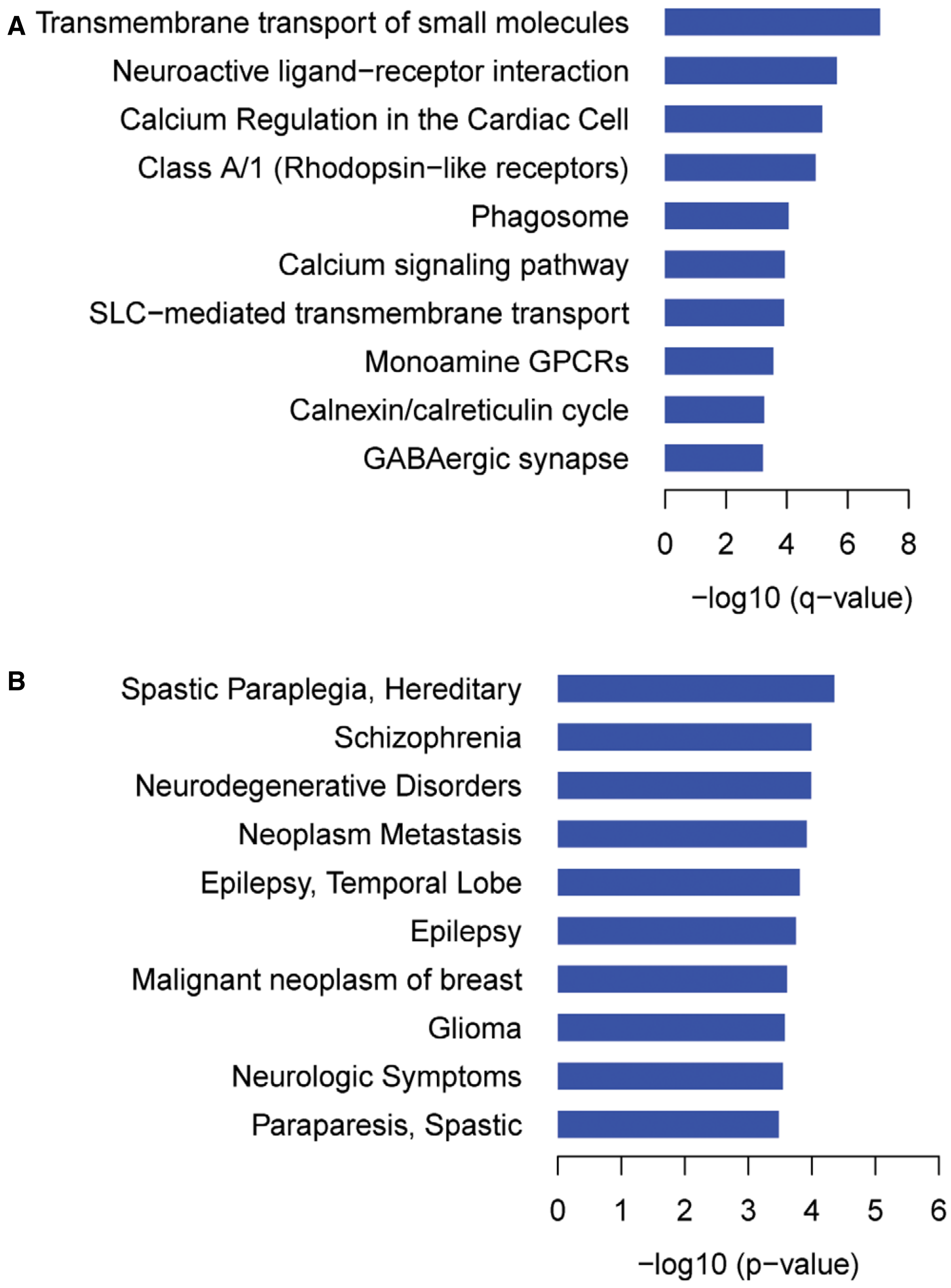

Figure EV2. Pathway enrichment analysis across baits and preys in MYTH GPCR interactome, and disease enrichment among preys.

A Pathways significantly enriched among baits and preys.

B Most highly represented diseases among preys based on unadjusted *P*-values.

© 2017 The Authors

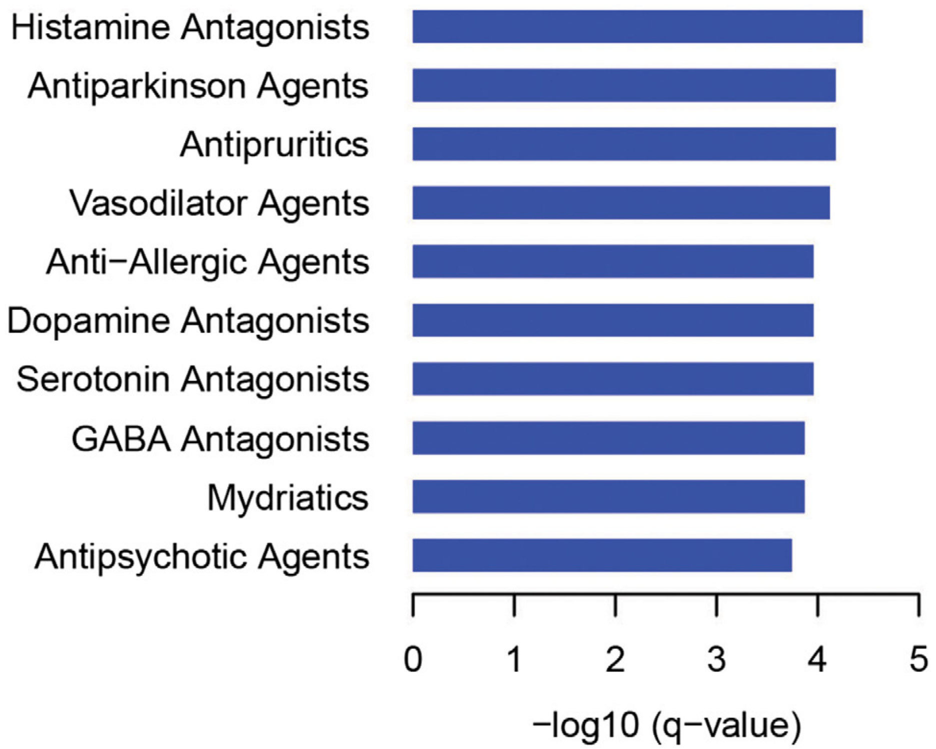

Figure EV3. Drug categories significantly enriched for bait and prey targets.

 © 2017 The Authors

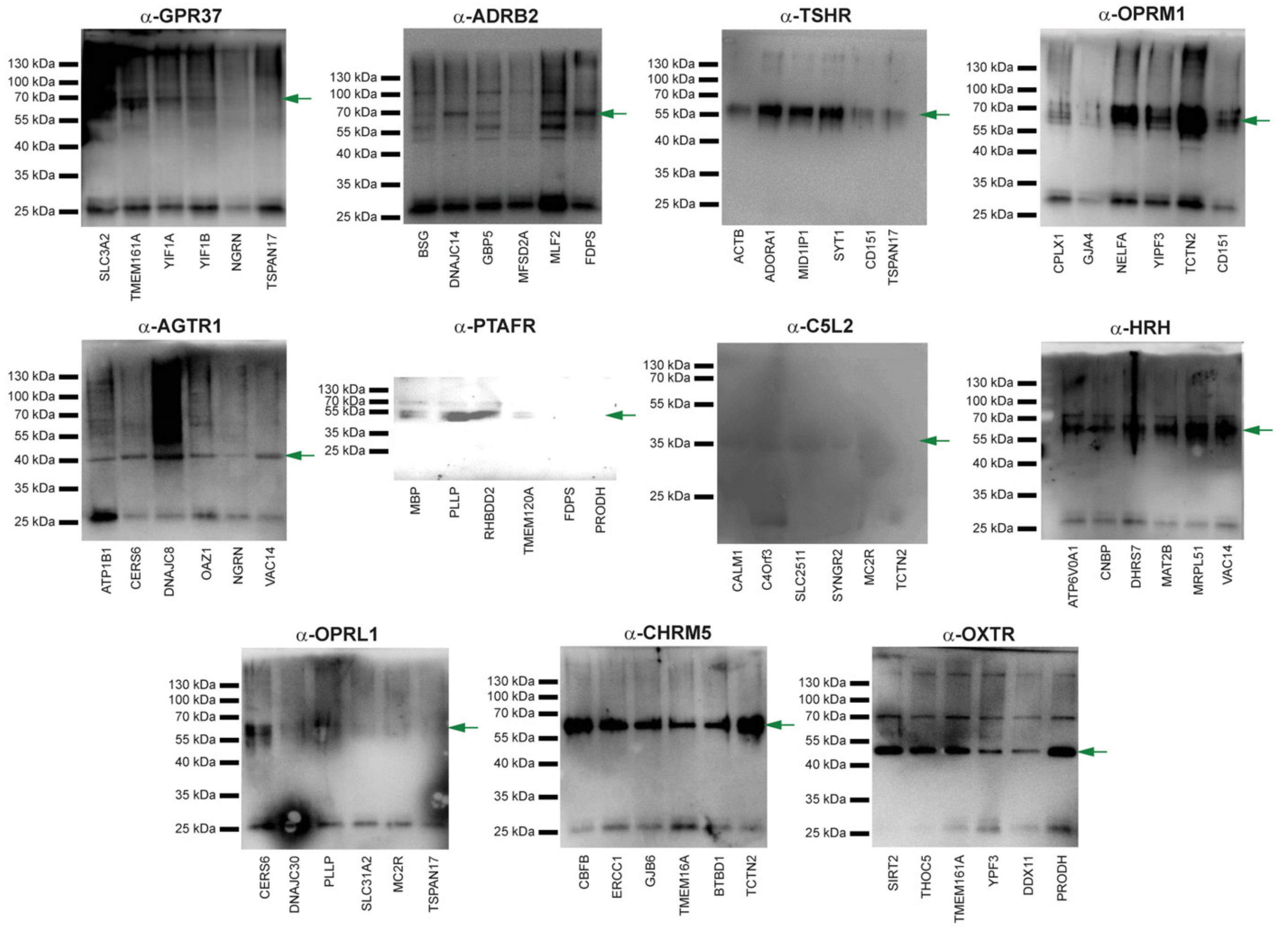

Figure EV4. Full Western blots of co-immunoprecipitation experiments used to orthogonally validate selected GPCR interactions.
Co-IPs were performed using α-FLAG antibody directed against overexpressed FLAG-tagged protein corresponding to either MYTH-identified interactor (first four lanes) or negative control (last two lanes), followed by Western blotting using antibody directed against the corresponding putative GPCR protein interaction partner (listed above each blot). Blots where both negative control samples interacted with a given GPCR were deemed non-specific under the test conditions used, preventing positive scoring of detected bands in the first four lanes. Green arrows denote the band corresponding to the GPCR protein indicated at top of each blot.

© 2017 The Authors

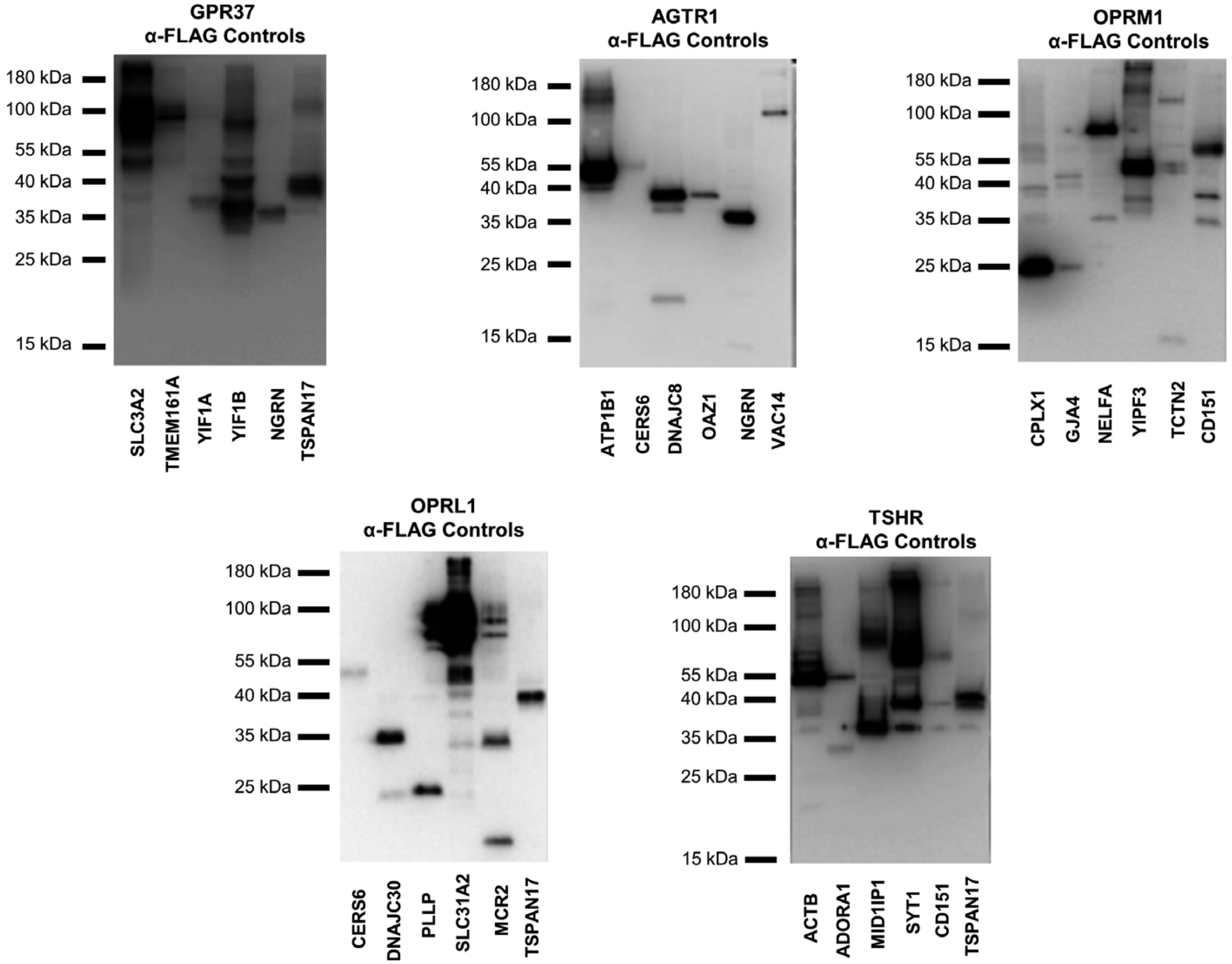

Figure EV5. Full blots showing expression of transiently transfected GPCR-interaction partners used in co-immunoprecipitation validation.

© 2017 The Authors

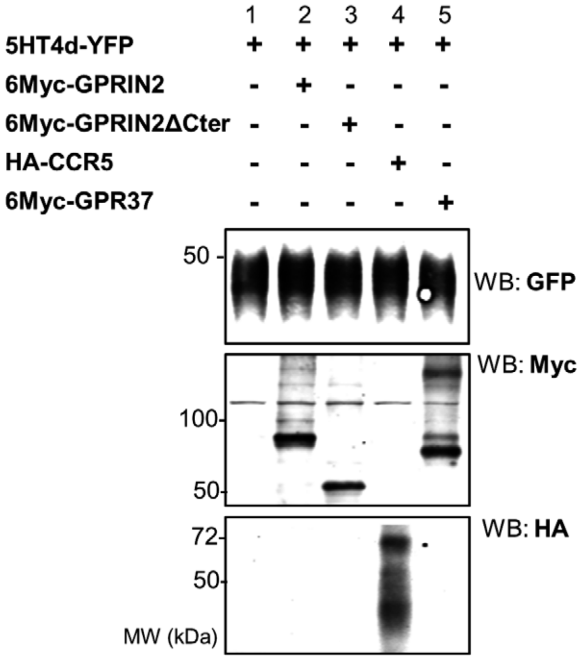

Figure EV6. Expression levels of transfected proteins monitored by Western blot.
No alteration in 5-HT4d level is observed in the presence of co-expressed GPRIN2, GPR37, and CCR5. Data are representative of at least two independent experiments.

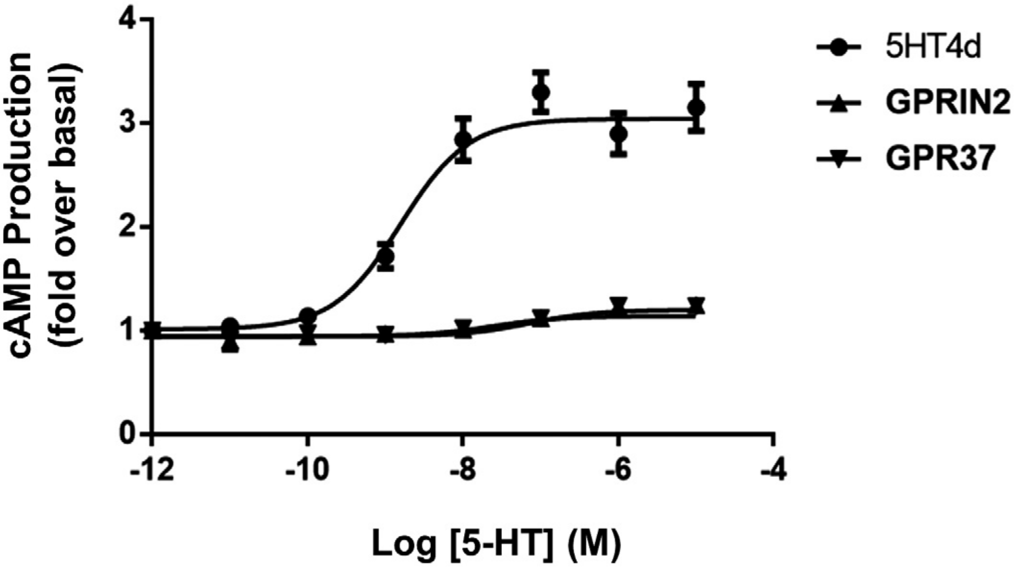

Figure EV7. Cyclic AMP levels in HEK-293 cells, in response to increasing concentrations of serotonin agonist and the presence of individually overexpressed 5-HT4d, GPRIN2, or GPR37.
Data are means of four independent measurements. Error bars indicate SEM.

© 2017 The Authors
