# Supplementary material for: Systematic protein–protein interaction mapping for clinically relevant human GPCRs
Source: Mol Syst Biol. 2017 Mar 15;13(3):918. doi: 10.15252/msb.20167430 (PMC5371730; doi:10.15252/msb.20167430)
Supplement: Supplementary file 2 — Table EV1 [file MSB-13-918-s002.docx]

**Table EV1. 48 GPCR baits and associated human diseases.**

| **Bait name** | **UniProt** | **Name** | **UniProt Alt. names** | **Class** | **Human Disease** | **Source** |
| --- | --- | --- | --- | --- | --- | --- |
| **HTR2C** | P28335 | 5-HT receptor , serotonin, 5-hydroxytryptamine | 5HT2C | A | Anorexia Nervosa; Behavior Disease; Bipolar Disorder; Hyperkinesis; Hypertension; Metabolic Syndrome X; ; Migraine Disorders; Obesity; Substance Withdrawal Syndrome; Weight Gain | OMIM |
| **GPR182** | O15218 | adrenomedullin receptor | ADMR, GPR182 | A | Adenoma; Pulmonary Disease, Chronic Obstructive; Systemic Infection | OMIM |
| **ADORA2A** | P29274 | adenosine A2A receptor | ADORA2A, ADORA2 | A | Amphetamine-Related Disorders; Anxiety Disorders; Asthma; Cardiomyopathies; Cystic Fibrosis; Drug Hypersensitivity; Drug Toxicity; Genetic Predisposition to Disease; Heart Failure; Huntington Disease; Hyperkinesis; Hypertension; Hypotension; Liver Cirrhosis, Experimental; Panic Disorder; Phobic Anxiety Disorder; Psychoses, Substance-Induced; Pulmonary Disease, Chronic Obstructive; Renal Insufficiency; Schizophrenia; Seizures; Sleep Initiation and Maintenance Disorders; Substance Withdrawal Syndrome; Thrombocytosis; Tremor; Ventricular Dysfunction | OMIM |
| **ADRA1A** | P35348 | alpha-1A adrenergic receptor | ADRA1A, ADRA1A | A | Diabetes Mellitus; Experimental; Hypertension; Liver Cirrhosis; Prostatic Neoplasms; Renal Insufficiency; Ventricular Remodeling | OMIM |
| **ADRB2** | P07550 | beta-2 adrenergic receptor | ADRB2, B2AR | A | Anemia; Sickle Cell; Arthritis; Arthritis, Rheumatoid; Asthma; Atherosclerosis; Autistic Disorder; Bronchial Diseases; Cardiomyopathy, Dilated; Cystic Fibrosis; Dermatitis; Diabetes Mellitus; Glaucoma; Graves Disease; Heart Failure; Hyperlipidemias; Hypertension; Hypokalemia; Insulin Resistance; Lung Diseases; Lung Neoplasms; Malaria; Metabolism Disease; Muscular Diseases; Myocardial Ischemia; Nervous System Diseases; Obesity; Peripheral Nervous System Diseases; Polycystic Ovary Syndrome; Premature Birth; Prostatic Neoplasms; Pulmonary Disease, Chronic Obstructive; Subarachnoid Hemorrhage; Substance-Related Disorders; Tremor | OMIM |
| **AGTR1** | P30556 | Angiotensin II receptor, type 1 | AGTR1, AGTR1A, AGTR1B, AT2R1, AT2R1B | A | Abortion; Adenocarcinoma; Adenoma; Adrenal Gland Tumor; Allanson Pantzar McLeod syndrome; Arthritis, Rheumatoid; Atherosclerosis; Brain Ischemia; Breast Neoplasms; Carcinoma, Pancreatic Ductal; Cardiovascular Diseases; Depressive Disorder; Diabetes Mellitus; Diabetic Retinopathy; Ductus Arteriosus, Patent; Embryoma; Esotropia; Glomerulonephritis, IGA; Heart Diseases; Heart Failure; Hyperaldosteronism; Hypercholesterolemia; Hypertension; Hypertension, Essential; Ischemia; Kidney Diseases; Lupus Erythematosus; Lupus Vulgaris; Mucocutaneous Lymph Node Syndrome; Neoplasm Metastasis; Obesity; Ovarian Neoplasms; Pituitary Neoplasms; Premature Birth; Proteinuria; Pulmonary Fibrosis; Renal Tubular Dysgenesis; Scleroderma, Systemic; Stomach Neoplasms | OMIM |
| **APLNR** | P35414 | apelin receptor | APJ, APLNR, AGTRL1 | A | Brain Neoplasms; Pre-Eclampsia; Stroke | OMIM |
| **BDKRB1** | P46663 | Bradykinin receptor B1 | BDKRB1, BRADYB1 | A | Adenoma; Asthma; Atopic Rhinitis; Diabetes Mellitus, Experimental; Epilepsy, Temporal Lobe; Heart Failure; Hyperalgesia; Inflammation; Multiple Sclerosis; Pain; Peptic Ulcer; Splenomegaly; Stomach Neoplasms | OMIM |
| **BDKRB2** | P30411 | Bradykinin receptor B2 | BDKRB2, BKR2 | A | Alzheimer Disease; Asthma; Cardiomyopathies; Epilepsy, Temporal Lobe; Extravasation of Diagnostic and Therapeutic Materials; Hemorrhage; Hyperalgesia; Hyperemia; Hypertension; Hypotension; Inflammation; Kidney Diseases; Obesity; Pain; Peptic Ulcer; Stomach Neoplasms; Thrombosis | OMIM |
| **GPR77** | Q9P296 | C5a anaphylatoxin chemotactic receptor | C5L2, GPR77 | A | Systemic Infection | OMIM |
| **CCR1** | P32246 | C-C chemokine receptor type 1 | CCR1, CMKBR1, CMKR1, SCYAR1 | A | Asthma; Carcinoma, Hepatocellular; Endometriosis; Hepatitis B; Hepatitis C; Liver Diseases; Liver Neoplasms; Neoplasm Metastasis; Neoplasms, Squamous Cell; Prostatic Neoplasms | OMIM |
| **CCR2** | P41597 | chemokine (C-C motif) receptor 2 | CCR2, CMKBR2 | A | Dermatitis; Allergic Contact; Drug-Induced Liver Injury; Liver Diseases; Pulmonary Fibrosis; Status Epilepticus | OMIM |
| **CCR4** | P51679 | chemokine (C-C motif) receptor 4 | CCR4, CMKBR4 | A | Celiac Disease; Dermatitis; Glioma; Leukemia; Liver Neoplasms; Lymphoma | OMIM |
| **CCR5** | P51681 | chemokine (C-C motif) receptor 5 | CCR5, CMKBR5 | A | Adenoviridae Infections; Alzheimer Disease; Aortic Aneurysm; Arthritis; Asthma; Atherosclerosis; Behcet Syndrome; Breast Neoplasms; Brucellosis; Chronic Rejection Of Renal Transplant; Colonic Neoplasms; Dermatitis, Atopic; Diabetes Mellitus; Diabetes Mellitus, Type 1; Encephalitis; Fetal Diseases; Glomerulonephritis, IGA; Graves Disease; Heart Failure; Hemophilia A; Hepatitis C; Hyperlipidemias; Hypertension; Liver Diseases; Liver Neoplasms; Lung Neoplasms; Lupus Erythematosus; Melanoma; Mucocutaneous Lymph Node Syndrome; Multiple Myeloma; Multiple Sclerosis; Neuritis; Polymyositis; Prostatic Neoplasms; Pulmonary Disease, Chronic Obstructive; Renal Insufficiency; Substance-Related Disorders; Testicular Dysfunction; Uterine Cervical Neoplasms; Viremia; West Nile Fever | OMIM |
| **CCR6** | P51684 | chemokine (C-C motif) receptor 6 | CCR6, CKRL3, CMKBR6, GPR29, STRL22 | A | Arthritis; Rheumatoid; Colitis, Ulcerative; Vitiligo | OMIM |
| **CCR8** | P51685 | chemokine (C-C motif) receptor 8 | CCR8, CKRL1, CMKBR8, CMKBRL2 | A | Status Epilepticus | OMIM |
| **CCR9** | P51686 | chemokine (C-C motif) receptor 9 | CCR9, GPR28 | A | Asthma; Celiac Disease; Lymphoproliferative Disorders; Melanoma; Neoplasm Metastasis; Prostatic Neoplasms; Status Epilepticus | OMIM |
| **CYSLTR2** | Q9NS75 | Cysteinyl leukotriene receptor 2 | CYSLTR2, CYSLT2, CYSLT2R | A | Asthma; Asthma, Aspirin-Induced; Drug Hypersensitivity; Pulmonary Fibrosis | OMIM |
| **DRD2** | P14416 | Dopamine receptor D2 | D2LR, DRD2 | A | Amphetamine-Related Disorders; Anxiety Disorders; Attention Deficit Disorder with Hyperactivity; Basal Ganglia Diseases; Bradycardia; Catalepsy; Cocaine-Related Disorders; Cognition Disorders; Dyskinesia, Drug-Induced; Dyskinesias; Heroin Dependence; Hyperkinesis; Hyperprolactinemia; Hypotension; Impulse Control Disorders; Movement Disorders; Myoclonic dystonia; Pain; Parkinsonian Disorders; Pituitary Neoplasms; Seizures; Substance-Related Disorders; Substance Withdrawal Syndrome; Tachycardia; Tobacco Use Disorder; Weight Gain | OMIM |
| **LPAR1** | Q92633 | Lysophosphatidic acid receptor 1 | EDG2, LPA1 | A | knee osteoarthritis | (Mototani *et al*, 2008) |
| **F2RL1** | P55085 | Protease activated receptor 2 (PAR2) | F2RL1, GPR11, PAR2 | A | Abortion; Adenocarcinoma, Papillary; Alzheimer Disease; Arthritis, Rheumatoid; Asthma; Atopic Rhinitis; Breast Neoplasms; Bronchopulmonary Dysplasia; Colonic Neoplasms; Dermatitis; Embryoma; Endometrial Neoplasms; Endometriosis; Gastroenteritis; Inflammation; Melanoma; Multiple Sclerosis; Neoplasm Metastasis; Pancreatic Neoplasms; Prostatic Neoplasms; Pruritus; Respiratory Insufficiency; Synovitis; Systemic Infection; Vascular Diseases | OMIM |
| **GNRHR** | P30968 | Gonadotropin-releasing hormone receptor | GNRHR, GRHR | A | Adrenal Gland Neoplasms; Heart Defects, Congenital; Hypogonadism | OMIM |
| **RXFP4** | Q8TDU9 | Relaxin-3 receptor 2 | GPR100, RXFP4, RLN3R2 | A | metabolic disturbance observed in patients treated with antipsychotics | (Munro *et al*, 2012) |
| **GPR35** | Q9HC97 | G protein-coupled receptor 35 | GPR35 | A | noninsulin-dependent diabetes mellitus | (Horikawa *et al*, 2000) |
| **GPR37** | O15354 | G protein-coupled receptor 37 | GPR37 | A | Parkinson's disease | (Imai *et al*, 2002) |
| **HRH1** | P35367 | Histamine H1 receptor | HRH1 | A | Atopic Rhinitis; Colonic Neoplasms; Hypotension; Infertility; Pain; Parkinson Disease; Respiratory Hypersensitivity; Rhinitis; Substance Withdrawal Syndrome | OMIM |
| **HTR2B** | P41595 | 5-hydroxytryptamine (serotonin) receptor 2B | HTR2B | A | Cardiomegaly; Colonic Neoplasms; Drug Toxicity; Heart Failure; Heart Valve Diseases; Hypertension; Hypertension, Pulmonary; Substance-Related Disorders | OMIM |
| **HTR4** | Q13639 | 5-hydroxytryptamine receptor 4 | HTR4 | A | Adenoviridae Infections; Adrenal Hyperplasia, Congenital; Anorexia; Arrhythmias, Cardiac; Atrial Fibrillation; Attention Deficit Disorder with Hyperactivity; Breast Neoplasms; Hypertension; Stroke; Tachycardia | OMIM |
| **HTR6** | P50406 | 5-HT6 receptor | HTR6 | A | Amphetamine-Related Disorders; Hypertension; Memory Disorders; Psychoses, Substance-Induced; Psychotic Disorders | OMIM |
| **LTB4R2** | Q9NPC1 | Leukotriene B4 receptor 2 | LTB4R2, BLT2R, BLTR2 | A | Arthritis; Rheumatoid; Ovarian Neoplasms | OMIM |
| **CHRM2** | P08172 | muscarinic acetylcholine receptor M2 | M2, CHRM2 | A | Behavior Disease; Bipolar Disorder; Bradycardia; Central Nervous System Disease; Depressive Disorder; Diabetes Mellitus, Experimental; Diabetes Mellitus, Type 1; Diabetic Neuropathies; Epilepsy; Substance-Related Disorders; Urinary Bladder Neoplasms; Urinary Bladder, Neurogenic | OMIM |
| **CHRM3** | P20309 | muscarinic acetylcholine receptor M3 | M3, CHRM3 | A | Ascites; Asthma; Diabetes Mellitus; Drug-Induced Liver Injury; Liver Cirrhosis; Neuroblastoma | OMIM |
| **CHRM4** | P08173 | muscarinic acetylcholine receptor M4 | M4, CHRM4 | A | Catatonia; Supranuclear Palsy, Progressive; Tremor | OMIM |
| **CHRM5** | P08912 | muscarinic acetylcholine receptor M5 | M5, CHRM5 | A | Cocaine-Related Disorders; Substance Withdrawal Syndrome | OMIM |
| **MC4R** | P32245 | Melanocortin receptor 4 | MC4R | A | Abortion; Body Weight; Bulimia; Embryoma; Hyperinsulinism; Insulin Resistance; Malnutrition; Obesity; Polycystic Ovary Syndrome; Spasms, Infantile | OMIM |
| **NPSR1** | Q6W5P4; Q56H78 | neuropeptide S receptor (NPSR) | NPSR1, GPR154, GPRA, PGR14 | A | Asthma; Asthma-Related Traits, Susceptibility To, 2; Bronchial Hyperreactivity; Dermatitis; Panic Disorder; Respiratory Insufficiency | OMIM |
| **OPRL1** | P41146 | nociceptin receptor | OPRL1, OOR, ORL1 | A | Arrhythmias; Cardiac; Hypokinesia; Morphine Dependence; Substance Withdrawal Syndrome | OMIM |
| **OXTR** | P30559 | oxytocin receptor | OXTR | A | Autistic Disorder; Carcinoma, Non-Small-Cell Lung; Child Development Disorders, Pervasive; Chromosome 3, monosomy 3p25; Cognition Disorders; Endometriosis; Heart Defects, Congenital; Leiomyoma | OMIM |
| **PTAFR** | P25105 | platelet-activating factor receptor | PTAFR, PAFR | A | allergy, asthma, septic shock, arterial thrombosis, and inflammatory processes | OMIM |
| **PTGER4** | P35408 | Prostaglandin E receptor 4 | PTGER4, PTGER2 | A | Arthritis; Experimental; Chloracne; Endometriosis; Inflammation; Lymphoma; Spondylitis, Ankylosing | OMIM |
| **RXFP3** | Q9NSD7 | Relaxin-3 receptor 1 | SALPR, RLN3R1, RXFP3 | A | (unknown) | OMIM |
| **TAAR1** | Q96RJ0 | Trace amine-associated receptor 1 | TAAR1, TA1, TAR1, TRAR1 | A | Depression; Schizophrenia | OMIM |
| **TSHR** | P16473 | thyrotropin receptor | TSHR, LGR3 | A | Abortion; Adenoma; Adrenal Gland Neoplasms; Carcinoma; Congenital Hypothyroidism; Graves Disease; Hyperthyroidism; Hyperthyroidism, Familial Gestational; Hyperthyroidism, Nonautoimmune; Lung Neoplasms; Lymphatic Metastasis; Rabies; Thyroid Diseases; Thyroid Neoplasms; Thyroiditis, Autoimmune; Thyrotoxicosis | OMIM |
| **OPRM1** | P35372 | μ-opioid receptors (MOR) | MOR1, OPRM1 | A | Adrenal Gland Tumor; Hemorrhage; Memory Disorders; Morphine Dependence; Subarachnoid Hemorrhage; Tremor | OMIM |
| **VIPR2** | P41587 | Vasoactive intestinal peptide receptor 2 | VIPR2, VIP2R | B | Arthritis; Arthritis, Rheumatoid; Gallbladder Disease 1; Gallbladder Diseases; Infection; Multiple Sclerosis | OMIM |
| **GPRC5B** | Q9NZH0; O75205 | retinoic acid-induced gene 2 protein | GPRC5B, RAIG2 | B | Obesity | OMIM |
| **FZD7** | O75084 | Frizzled | FZD7 | Frizzled | Breast Neoplasms; | OMIM |
| **SMO** | Q99835 | Smoothened | SMO, SMOH | Frizzled | Bone Diseases; Chondrosarcoma; Hyperglycemia; Jaw Abnormalities; Pancreatic Neoplasms; Skin Neoplasms | OMIM |

**REFERENCES**

Horikawa Y, Oda N, Cox NJ, Li X, Orho-Melander M, Hara M, Hinokio Y, Lindner TH, Mashima H, Schwarz PE, del Bosque-Plata L, Horikawa Y, Oda Y, Yoshiuchi I, Colilla S, Polonsky KS, Wei S, Concannon P, Iwasaki N, Schulze J, et al (2000) Genetic variation in the gene encoding calpain-10 is associated with type 2 diabetes mellitus. *Nat. Genet.* **26:** 163–75 Available at: http://www.ncbi.nlm.nih.gov/pubmed/11017071 [Accessed October 13, 2016]

Imai Y, Soda M, Hatakeyama S, Akagi T, Hashikawa T, Nakayama KI & Takahashi R (2002) CHIP is associated with Parkin, a gene responsible for familial Parkinson’s disease, and enhances its ubiquitin ligase activity. *Mol. Cell* **10:** 55–67 Available at: http://www.ncbi.nlm.nih.gov/pubmed/12150907 [Accessed October 13, 2016]

Mototani H, Iida A, Nakajima M, Furuichi T, Miyamoto Y, Tsunoda T, Sudo A, Kotani A, Uchida A, Ozaki K, Tanaka Y, Nakamura Y, Tanaka T, Notoya K & Ikegawa S (2008) A functional SNP in EDG2 increases susceptibility to knee osteoarthritis in Japanese. *Hum. Mol. Genet.* **17:** 1790–7 Available at: http://www.ncbi.nlm.nih.gov/pubmed/18325907 [Accessed October 13, 2016]

Munro J, Skrobot O, Sanyoura M, Kay V, Susce MT, Glaser PEA, de Leon J, Blakemore AIF & Arranz MJ (2012) Relaxin polymorphisms associated with metabolic disturbance in patients treated with antipsychotics. *J. Psychopharmacol.* **26:** 374–9 Available at: http://www.ncbi.nlm.nih.gov/pubmed/21693553 [Accessed October 13, 2016]
