# Supplementary material for: Systematic protein–protein interaction mapping for clinically relevant human GPCRs
Source: Mol Syst Biol. 2017 Mar 15;13(3):918. doi: 10.15252/msb.20167430 (PMC5371730; doi:10.15252/msb.20167430)
Supplement: Supplementary file 5 — Table EV4 [file MSB-13-918-s005.docx]

**Table EV4. Spurious prey proteins identified during MYTH screening.**

| **Prey Uniprot** | **Prey Accession** | **Prey Name** | **Reason for removal** |
| --- | --- | --- | --- |
| O75503 | NM_006493.2 | Homo sapiens ceroid-lipofuscinosis, neuronal 5 (CLN5), mRNA | Signal peptide processing |
| P09958 | NM_002569.2 | Homo sapiens furin (paired basic amino acid cleaving enzyme) (FURIN), mRNA | Signal peptide processing |
| Q86WA8 | NM_031490.2 | Homo sapiens lon peptidase 2, peroxisomal (LONP2), mRNA | Signal peptide processing |
| Q92824 | NM_006200.3 | Homo sapiens proprotein convertase subtilisin/kexin type 5 (PCSK5), transcript variant 2, mRNA | Signal peptide processing |
| H0YK72 |  | Homo sapiens SEC11 homolog A (S. cerevisiae) (SEC11A), mRNA | Signal peptide processing |
| H0YK83 |  | Homo sapiens SEC11 homolog A (S. cerevisiae) (SEC11A), mRNA | Signal peptide processing |
| H0YKT4 |  | Homo sapiens SEC11 homolog A (S. cerevisiae) (SEC11A), mRNA | Signal peptide processing |
| H0YNA5 |  | Homo sapiens SEC11 homolog A (S. cerevisiae) (SEC11A), mRNA | Signal peptide processing |
| H0YNG3 |  | Homo sapiens SEC11 homolog A (S. cerevisiae) (SEC11A), mRNA | Signal peptide processing |
| H0YNX5 |  | Homo sapiens SEC11 homolog A (S. cerevisiae) (SEC11A), mRNA | Signal peptide processing |
| P67812 | NM_014300.2 | Homo sapiens SEC11 homolog A (S. cerevisiae) (SEC11A), mRNA | Signal peptide processing |
| B4DUL4 |  | Homo sapiens SEC11 homolog A (S. cerevisiae) (SEC11A), mRNA | Signal peptide processing |
| B4DI03 |  | Homo sapiens SEC11 homolog C (S. cerevisiae) (SEC11C), mRNA | Signal peptide processing |
| Q9BY50 | NM_033280.2 | Homo sapiens SEC11 homolog C (S. cerevisiae) (SEC11C), mRNA | Signal peptide processing |
| C9JBL1 |  | Homo sapiens signal peptidase complex subunit 1 homolog (S. cerevisiae) (SPCS1), mRNA | Signal peptide processing |
| Q9Y6A9 | NM_014041.3 | Homo sapiens signal peptidase complex subunit 1 homolog (S. cerevisiae) (SPCS1), mRNA | Signal peptide processing |
| E9PI68 |  | Homo sapiens signal peptidase complex subunit 2 homolog (S. cerevisiae) (SPCS2), mRNA | Signal peptide processing |
| E9PL01 |  | Homo sapiens signal peptidase complex subunit 2 homolog (S. cerevisiae) (SPCS2), mRNA | Signal peptide processing |
| E9PRB9 |  | Homo sapiens signal peptidase complex subunit 2 homolog (S. cerevisiae) (SPCS2), mRNA | Signal peptide processing |
| H0YE04 |  | Homo sapiens signal peptidase complex subunit 2 homolog (S. cerevisiae) (SPCS2), mRNA | Signal peptide processing |
| Q15005 | NM_014752.2 | Homo sapiens signal peptidase complex subunit 2 homolog (S. cerevisiae) (SPCS2), mRNA | Signal peptide processing |
| P61009 | NM_021928.3 | Homo sapiens signal peptidase complex subunit 3 homolog (S. cerevisiae) (SPCS3), mRNA | Signal peptide processing |
| P0C7V7 |  | Putative signal peptidase complex catalytic subunit SEC11B | Signal peptide processing |
|  | NR_003286.1 | Homo sapiens 18S ribosomal RNA (LOC100008588) | Ribosomal contamination |
|  | NM_203372.1 | Homo sapiens acyl-CoA synthetase long-chain family member 3 (ACSL3), transcript variant 2, mRNA | Ribosomal contamination |
|  | NM_022977.1 | Homo sapiens acyl-CoA synthetase long-chain family member 4 (ACSL4), transcript variant 2, mRNA | Ribosomal contamination |
|  | NM_005469.2 | Homo sapiens acyl-CoA thioesterase 8 (ACOT8), transcript variant 1, mRNA | Ribosomal contamination |
|  | NM_004371.3 | Homo sapiens coatomer protein complex, subunit alpha (COPA), transcript variant 2, mRNA | Ribosomal contamination |
|  | NM_001402.5 | Homo sapiens eukaryotic translation elongation factor 1 alpha 1 (EEF1A1), mRNA | Ribosomal contamination |
|  | NM_001404.4 | Homo sapiens eukaryotic translation elongation factor 1 gamma (EEF1G), mRNA | Ribosomal contamination |
|  | NM_005345.4 | Homo sapiens heat shock 70kDa protein 1A (HSPA1A), mRNA | Ribosomal contamination |
|  | NM_005347.2 | Homo sapiens heat shock 70kDa protein 5 (glucose-regulated protein, 78kDa) (HSPA5), mRNA | Ribosomal contamination |
|  | NM_006597.3 | Homo sapiens heat shock 70kDa protein 8 (HSPA8), transcript variant 1, mRNA | Ribosomal contamination |
|  | NM_153201.1 | Homo sapiens heat shock 70kDa protein 8 (HSPA8), transcript variant 2, mRNA | Ribosomal contamination |
|  | NM_004134.5 | Homo sapiens heat shock 70kDa protein 9 (mortalin) (HSPA9), nuclear gene encoding mitochondrial protein, mRNA | Ribosomal contamination |
|  | NM_003512.3 | Homo sapiens histone cluster 1, H2ac (HIST1H2AC), mRNA | Ribosomal contamination |
|  | NM_000421.2 | Homo sapiens keratin 10 (epidermolytic hyperkeratosis; keratosis palmaris et plantaris) (KRT10), mRNA | Ribosomal contamination |
|  | NM_033061.2 | Homo sapiens keratin associated protein 4-7 (KRTAP4-7), mRNA | Ribosomal contamination |
|  | NM_020236.3 | Homo sapiens mitochondrial ribosomal protein L1 (MRPL1), nuclear gene encoding mitochondrial protein, mRNA | Ribosomal contamination |
|  | NM_032478.2 | Homo sapiens mitochondrial ribosomal protein L38 (MRPL38), nuclear gene encoding mitochondrial protein, mRNA | Ribosomal contamination |
|  | NM_014046.2 | Homo sapiens mitochondrial ribosomal protein S18B (MRPS18B), nuclear gene encoding mitochondrial protein, mRNA | Ribosomal contamination |
|  | NM_199177.1 | Homo sapiens mitochondrial ribosome recycling factor (MRRF), nuclear gene encoding mitochondrial protein, transcript variant 2, mRNA | Ribosomal contamination |
|  | NM_020967.2 | Homo sapiens nuclear receptor coactivator 5 (NCOA5), mRNA | Ribosomal contamination |
|  | NM_001099335.1 | Homo sapiens phytanoyl-CoA 2-hydroxylase interacting protein (PHYHIP), transcript variant 1, mRNA | Ribosomal contamination |
|  | NM_014759.3 | Homo sapiens phytanoyl-CoA 2-hydroxylase interacting protein (PHYHIP), transcript variant 2, mRNA | Ribosomal contamination |
|  | NM_032439.1 | Homo sapiens phytanoyl-CoA 2-hydroxylase interacting protein-like (PHYHIPL), mRNA | Ribosomal contamination |
|  | NM_012423.2 | Homo sapiens ribosomal protein L13a (RPL13A), mRNA | Ribosomal contamination |
|  | NM_002948.2 | Homo sapiens ribosomal protein L15 (RPL15), mRNA | Ribosomal contamination |
|  | NM_002948.2 | Homo sapiens ribosomal protein L15 (RPL15), mRNA | Ribosomal contamination |
|  | NM_000983.3 | Homo sapiens ribosomal protein L22 (RPL22), mRNA | Ribosomal contamination |
|  | NM_000969.3 | Homo sapiens ribosomal protein L5 (RPL5), mRNA | Ribosomal contamination |
|  | NM_000971.3 | Homo sapiens ribosomal protein L7 (RPL7), mRNA | Ribosomal contamination |
|  | NM_002952.3 | Homo sapiens ribosomal protein S2 (RPS2), mRNA | Ribosomal contamination |
|  | NM_001031.4 | Homo sapiens ribosomal protein S28 (RPS28), mRNA | Ribosomal contamination |
|  | NM_001007.4 | Homo sapiens ribosomal protein S4, X-linked (RPS4X), mRNA | Ribosomal contamination |
|  | NM_001002.3 | Homo sapiens ribosomal protein, large, P0 (RPLP0), transcript variant 1, mRNA | Ribosomal contamination |
|  | NM_001037582.2 | Homo sapiens stearoyl-CoA desaturase 5 (SCD5), transcript variant 1, mRNA | Ribosomal contamination |
|  | NM_003850.1 | Homo sapiens succinate-CoA ligase, ADP-forming, beta subunit (SUCLA2), mRNA | Ribosomal contamination |
|  | NM_001012979.1 | Homo sapiens transcription elongation factor A (SII)-like 5 (TCEAL5), mRNA | Ribosomal contamination |
|  | NM_001006938.2 | Homo sapiens transcription elongation factor A (SII)-like 6 (TCEAL6), mRNA | Ribosomal contamination |
|  | NM_003321.3 | Homo sapiens Tu translation elongation factor, mitochondrial (TUFM), nuclear gene encoding mitochondrial protein, mRNA | Ribosomal contamination |
|  | NM_004607.2 | Homo sapiens tubulin folding cofactor A (TBCA), mRNA | Ribosomal contamination |
|  | NM_001281.2 | Homo sapiens tubulin folding cofactor B (TBCB), mRNA | Ribosomal contamination |
|  | NM_007030.2 | Homo sapiens tubulin polymerization promoting protein (TPPP), mRNA | Ribosomal contamination |
|  | NM_015964.2 | Homo sapiens tubulin polymerization-promoting protein family member 3 (TPPP3), mRNA | Ribosomal contamination |
|  | NM_006082.2 | Homo sapiens tubulin, alpha 1b (TUBA1B), mRNA | Ribosomal contamination |
|  | NM_006000.1 | Homo sapiens tubulin, alpha 4a (TUBA4A), mRNA | Ribosomal contamination |
|  | NM_178012.3 | Homo sapiens tubulin, beta 2B (TUBB2B), mRNA | Ribosomal contamination |
|  | NM_006088.5 | Homo sapiens tubulin, beta 2C (TUBB2C), mRNA | Ribosomal contamination |
|  | NM_021821.3 | Homo sapiens mitochondrial ribosomal protein S35 (MRPS35), nuclear gene encoding mitochondrial protein, transcript variant 1, mRNA | Ribosomal contamination |
|  | NM_001281.2 | Homo sapiens tubulin folding cofactor B (TBCB), mRNA | Ribosomal contamination |
|  | NM_001769.3 | Homo sapiens CD9 molecule (CD9), mRNA | Non-specific interaction with unrelated bait in multiple screens |
|  | NM_001001994.1 | Homo sapiens glycoprotein M6B (GPM6B), transcript variant 4, mRNA | Non-specific interaction with unrelated bait in multiple screens |
|  | NM_002237.3 | Homo sapiens potassium voltage-gated channel, subfamily G, member 1 (KCNG1), mRNA | Non-specific interaction with unrelated bait in multiple screens |
|  | NM_181726.2 | Homo sapiens ankyrin repeat domain 37 (ANKRD37), mRNA | Non-specific interaction with unrelated bait in multiple screens |
|  | NM_017801.2 | Homo sapiens CKLF-like MARVEL transmembrane domain containing 6 (CMTM6), mRNA | Non-specific interaction with unrelated bait in multiple screens |
|  | NM_001040181.1 | Homo sapiens claudin domain containing 1 (CLDND1), transcript variant 1, mRNA | Non-specific interaction with unrelated bait in multiple screens |
|  | NM_201591.1 | Homo sapiens glycoprotein M6A (GPM6A), transcript variant 2, mRNA | Non-specific interaction with unrelated bait in multiple screens |
|  | NM_014644.4 | Homo sapiens phosphodiesterase 4D interacting protein (PDE4DIP), transcript variant 1, mRNA | Non-specific interaction with unrelated bait in multiple screens |
|  | NM_002237.3 | Homo sapiens potassium voltage-gated channel, subfamily G, member 1 (KCNG1), mRNA | Non-specific interaction with unrelated bait in multiple screens |
|  | NM_006423.2 | Homo sapiens Rab acceptor 1 (prenylated) (RABAC1), mRNA | Non-specific interaction with unrelated bait in multiple screens |
|  | NM_000986.3 | Homo sapiens ribosomal protein L24 (RPL24), mRNA | Non-specific interaction with unrelated bait in multiple screens |
|  | NM_194358.1 | Homo sapiens ring finger protein 41 (RNF41), transcript variant 2, mRNA | Non-specific interaction with unrelated bait in multiple screens |
|  | NM_018955.2 | Homo sapiens ubiquitin B (UBB), mRNA | Non-specific interaction with unrelated bait in multiple screens |
|  | NM_145062.2 | Homo sapiens zinc finger with UFM1-specific peptidase domain (ZUFSP), mRNA | Non-specific interaction with unrelated bait in multiple screens |
