# Supplementary figures and images for: Author Correction: Systematic protein-protein interaction mapping for clinically relevant human GPCRs
Source: Mol Syst Biol. 2025 Jan 6;21(2):208–9. doi: 10.1038/s44320-024-00080-3 (PMC11790839; doi:10.1038/s44320-024-00080-3)

## ADRA1A.pCCW-STE

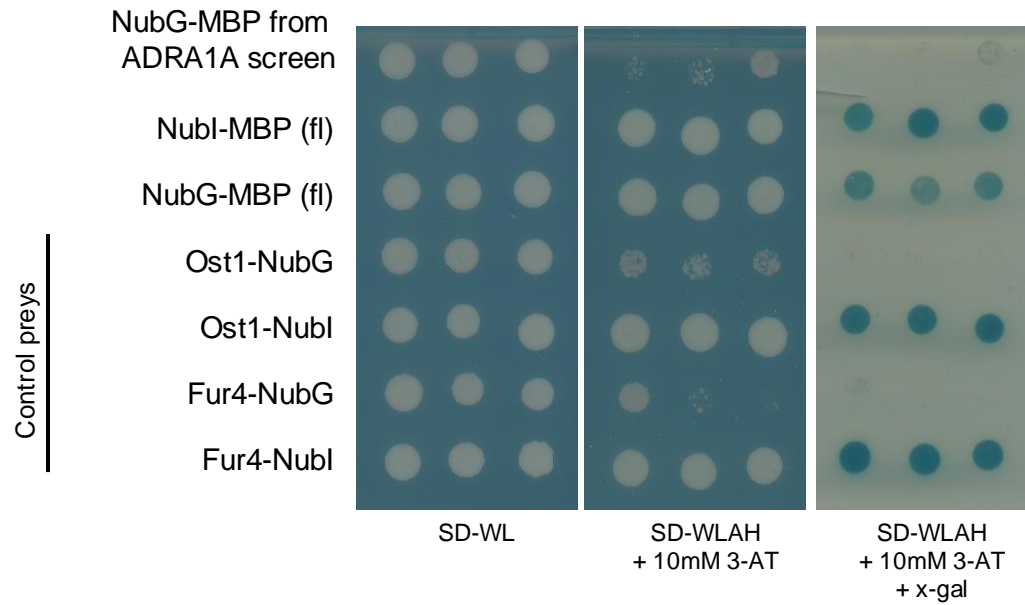

Supplement: Supplementary file 1 — Full ADR1A1 Sample Images on Plates [file 44320_2024_80_MOESM1_ESM.pdf]

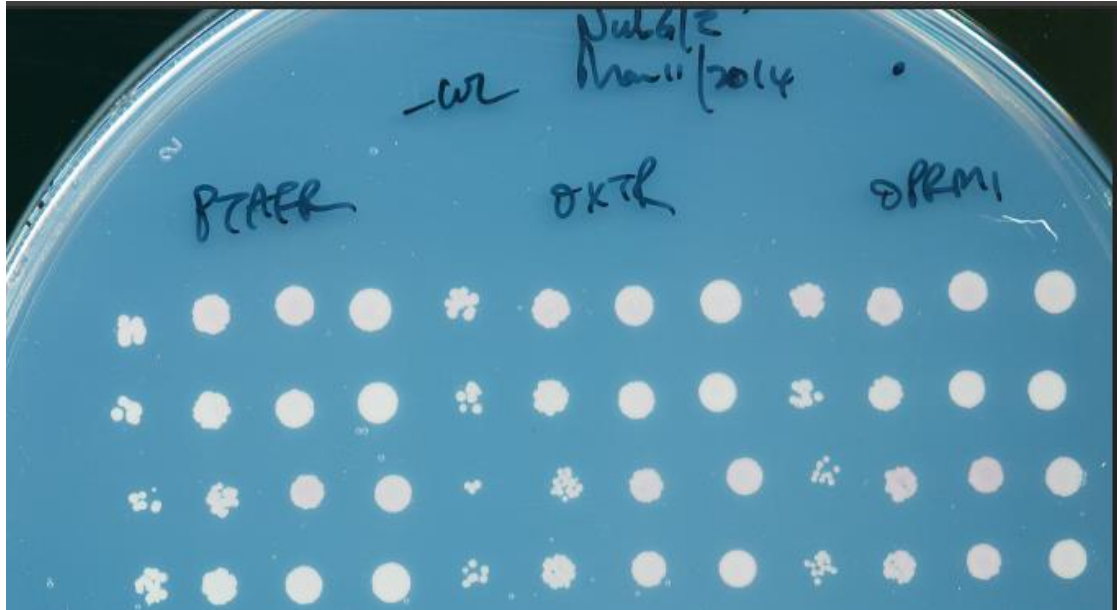

OPRM1 and PTAFR (-WL)

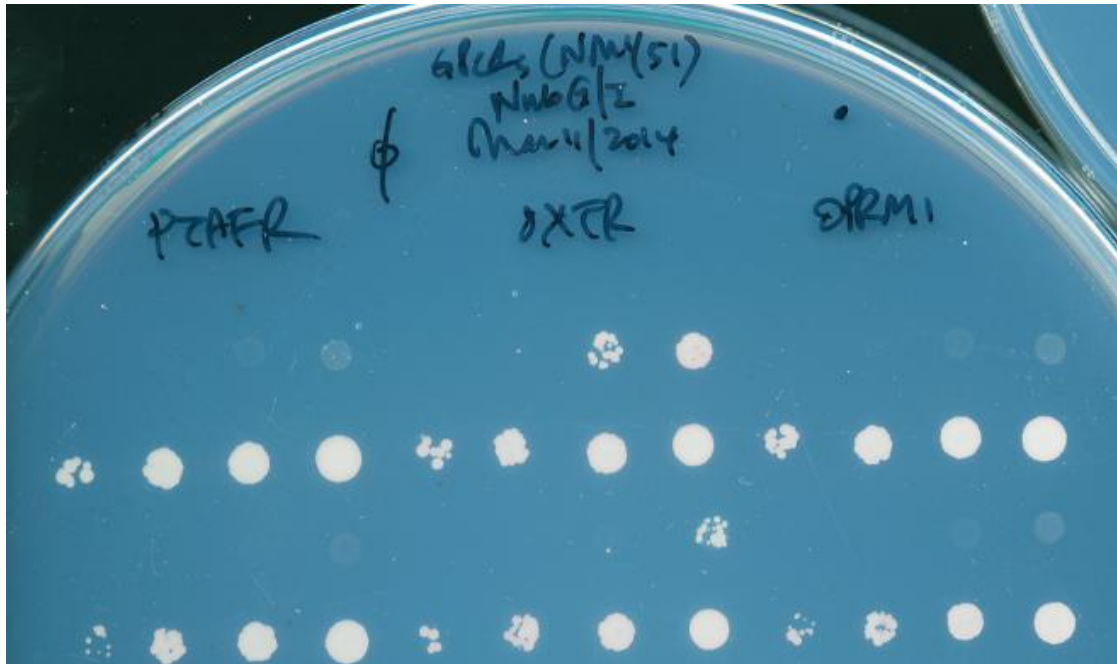

OPRM1 and PTAFR (-WLAH)

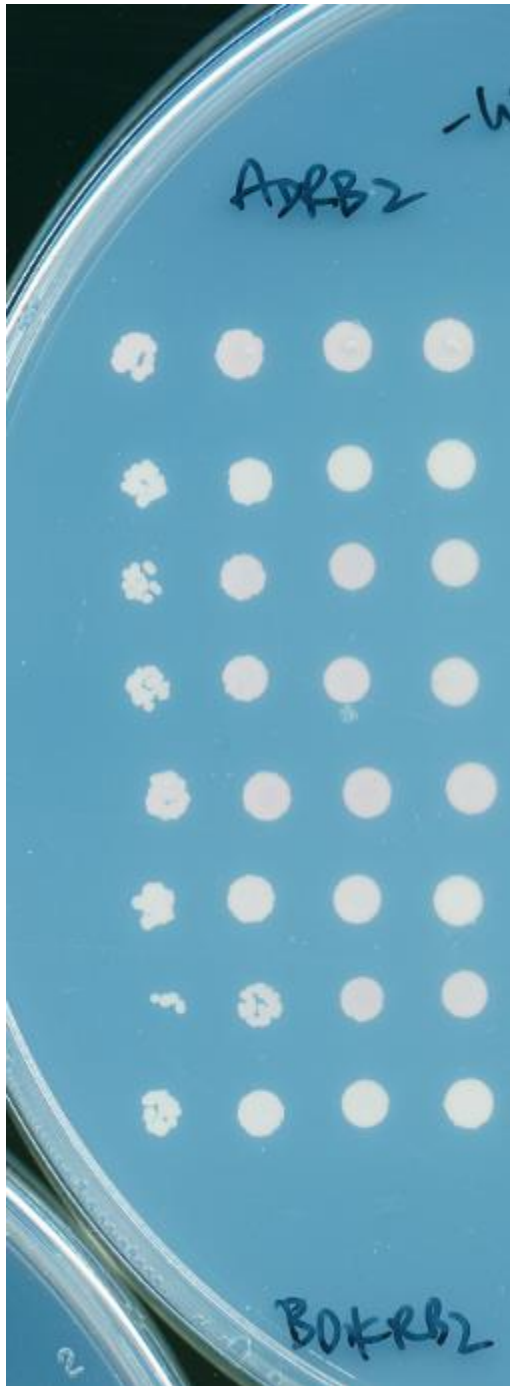

ADRB2 and  
BDKRB2 (-WL)

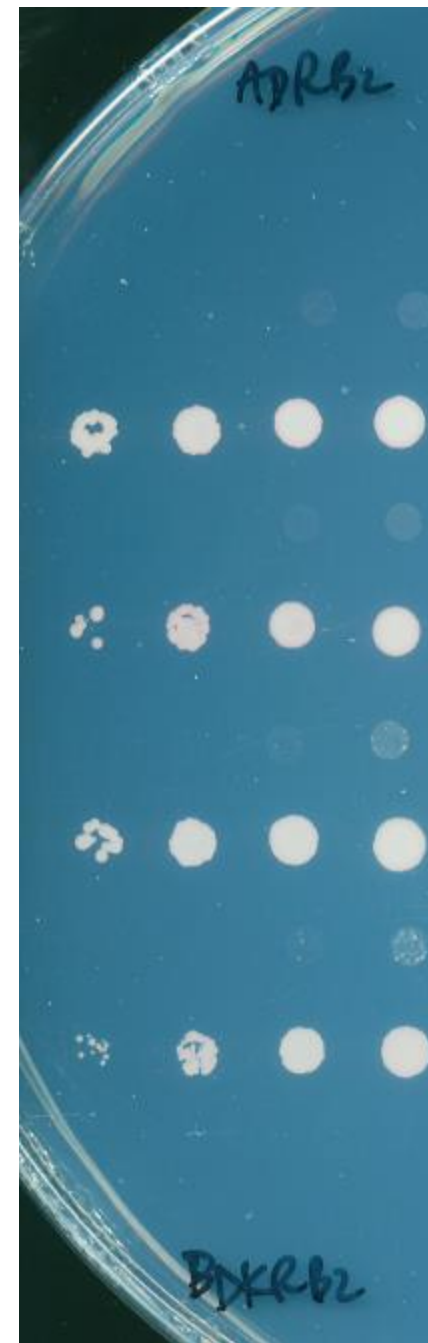

ADRB2 and  
BDKRB2 (-WLAH)

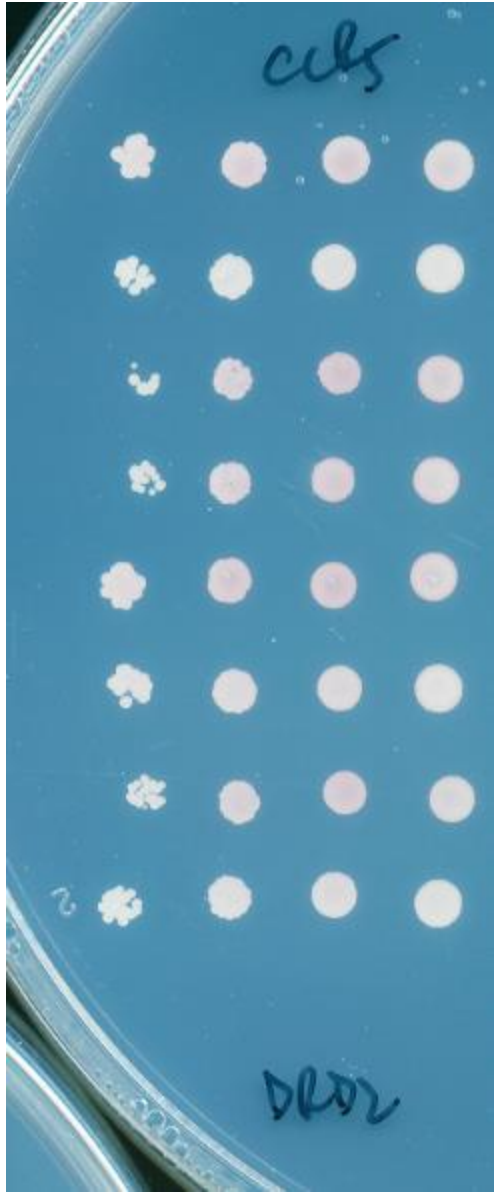

DRD2 (-WL)

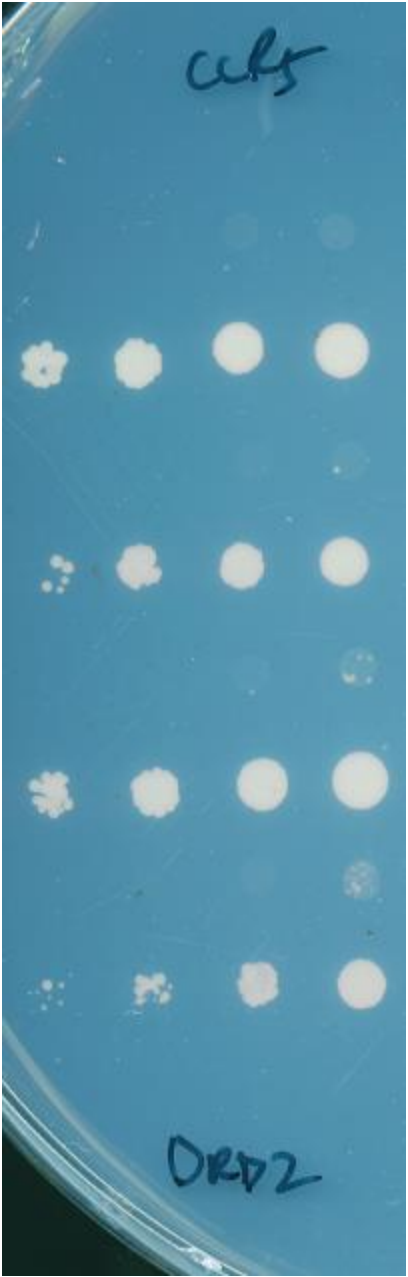

DRD2 (-WLAH)

Supplement: Supplementary file 2 — Source Plate Images [file 44320_2024_80_MOESM2_ESM.pdf]
